# Family history of cancer as a potential risk factor for colorectal cancer in EMRO countries: a systematic review and meta-analysis

**DOI:** 10.1038/s41598-023-44487-8

**Published:** 2023-10-14

**Authors:** Mohammad-Hossein Keivanlou, Ehsan Amini-Salehi, Farahnaz Joukar, Negin Letafatkar, Arman Habibi, Naeim Norouzi, Azin Vakilpour, Maryam Sadat Aleali, Zahra Rafat, Mohammad Taghi Ashoobi, Fariborz Mansour-Ghanaei, Soheil Hassanipour

**Affiliations:** 1 Gastrointestinal and Liver Diseases Research Center, Guilan University of Medical Sciences, Rasht, Iran; 2 Student Research Committee, School of Medicine, Guilan University of Medical Sciences, Rasht, Iran; 3 Guilan University of Medical Sciences, Rasht, Iran; 4 Department of Medical Parasitology and Mycology, School of Medicine, Guilan University of Medical Sciences, Rasht, Iran; 5 Razi Clinical Research Development Unit, Guilan University of Medical Sciences, Rasht, Iran

**Keywords:** Cancer, Gastroenterology, Risk factors

## Abstract

The current meta-analysis aims to investigate the existing articles that evaluated the implications of a positive family history of cancer on the risk of colorectal cancer (CRC) within the EMRO countries. We employed PubMed, Scopus, and Web of Science as search databases for this study. To assess the quality of the selected articles, we utilized the Newcastle–Ottawa (NCO) checklist. In comparing the impact of a family history of cancer between the case and control groups, we computed the odds ratio (OR) along with its corresponding 95% confidence interval (CI). Finally, 27 articles were selected for meta-analysis. The result of the meta-analysis showed a significant association between the presence of a family history of CRC or any cancers and CRC (OR 2.21; 95% CI 1.54–3.17; P < 0.001, OR 1.76; 95% CI 1.27–2.42; P = 0.001, respectively). Our findings underscore the critical importance of timely screening and early identification for individuals with a family history of cancer. By fostering close coordination among healthcare facilities and actively promoting the adoption of screening methods for early detection, we have the potential to significantly reduce both mortality rates and financial burdens of CRC on the general public, ultimately leading to enhanced patient outcomes.

## Introduction

Colorectal cancer (CRC) ranks as the third most frequently diagnosed cancer globally and is the second leading cause of cancer-related deaths worldwide. It constitutes approximately 10% of all cancer cases and is responsible for 9.4% of cancer-related fatalities on a global scale^1^. The incidence of CRC is higher in highly-developed countries, while the increasing number of CRC cases in middle- and low-income countries may be linked to the process of Westernization^2^. For example, in a recent analysis conducted by Mehta et al., it was found that adherence to Western dietary patterns was significantly associated with an increased risk of CRC^3^.

CRC is influenced by a combination of environmental and genetic factors. Hereditary syndromes, including Lynch syndrome (commonly referred as Hereditary Non-Polyposis Colorectal Cancer Syndrome or HNPCC) and familial adenomatous polyposis (also known as Familial Polyposis Coli), are associated with a significantly elevated risk of CRC^4–6^. Hereditary CRCs constitute around 5% of all CRC cases. While the risk of CRC in the general population is approximately 6%, individuals with a positive family history face a significantly higher risk. Those with a family history of CRC have a 2 to 4 times higher chance of CRC compared to individuals without a family history. This increased risk is attributed to the inheritance of mutant genes passed down from one generation to the next, underscoring the role of genetics in cancer development^7–9^. Previous studies reported that the risk for the development of CRC is approximately 1.8–1.9 times higher in patients with positive family history in a first-degree relative reflecting the need for more diagnostic approaches in these patients^10^.

The Eastern Mediterranean Region (EMRO), one of the six WHO regions, encompasses 22 countries with a total population of 730 million people. In this region, the incidence of CRC stands at approximately 5.9% in both men and women, making it the second most prevalent cause of cancer^11^. Furthermore, Nikbakht et al. demonstrated that the survival rate of CRC in the USA and European countries is higher compared to EMRO countries. This underscores the need for specific attention and targeted interventions in the EMRO region^12^.

Since individuals with a positive family history of cancer are classified as a high-risk group for developing CRC, early detection and clinical management are essential to enhance prognosis. Guidelines recommend increased screening interventions for these individuals to ensure timely identification and treatment^13,14^.

To recognizing the pivotal role of a positive family history in increasing CRC risk, it becomes imperative to determine the precise extent of this impact within the EMRO population. Conducting comprehensive epidemiological studies to evaluate the overall influence of this risk factor can offer invaluable insights into our understanding of the various global risk factors associated with CRC. While a positive family history of cancer has been suggested as a potential risk factor for CRC, its exact strength and significance remain uncertain especially in EMRO countries. In this meta-analysis study, we aimed to assess the impact of a positive family history of CRC or any cancers on the development of CRC in EMRO countries.

## Method

### Reporting

This systematic review and meta-analysis is conducted according to the guidelines of Preferred Reporting Items for Systematic Reviews and Meta-Analysis (PRISMA)^15^.

### Search strategy

We choose PubMed, Scopus, and Web of Science as our databases for a comprehensive systematic search by utilization of the following keyword: "Colorectal Tumors," "Colorectal Neoplasms," "Colorectal cancer," "Risk Factor," "Iran," "Afghanistan," "Bahrain," "Djibouti," "Egypt," "Iraq," "Jordan," "Kuwait," "Lebanon," "Libya," "Morocco," "Oman," "Pakistan," "State of Palestine," "Palestine," "Qatar," "Saudi Arabia," "Somalia," "Sudan," "Syria," "Tunisia," "United Arab Emirates," and "Yemen". We also examined the reference lists of the included studies. Our search extended up to December 2022.

### Study selection

To mitigate the risk of selective reporting bias, a panel of four independent researchers conducted a comprehensive review of all papers. Throughout the screening process, any disagreements among researchers regarding study selection were resolved with the input of the corresponding author.

### Data extraction procedure

The data extraction process was carried out by three independent researchers. We extracted all of the essential information from the included studies by designing a checklist with the following sections: First author's name, year of publication, country, sample size, base characteristics of patients (e.g., age, gender), and status of family history (CRC or any cancers). It is noticeable that we considered the positive family history of cancer in first-degree, second-degree, and third-degree families separately. In all stages of reviewing the articles, any conflict between the researchers was resolved with the final decision of the corresponding author.

### Inclusion and exclusion criteria

We established a set of criteria to meticulously assess all papers considered for inclusion in our study. Our inclusion criteria consisted of case–control studies conducted within EMRO countries. These studies were required to have at least one relevant data point regarding a family history of cancer in both the case and control groups, with a specific focus on examining its impact on CRC. Additionally, we restricted our selection to English-language literature.

Excluded from consideration were articles where control groups were drawn from sources other than CRC, such as colon polyps or colorectal adenomas. Studies that utilized healthy tissues as control groups were also excluded. Furthermore, we omitted studies that assessed both CRC and colon polyps within the case group.

### Quality assessment

**Figure 1:**
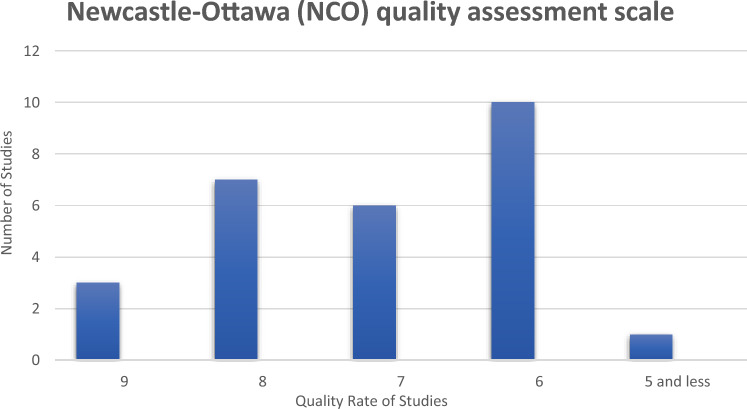
Newcastle–Ottawa (NCO) quality assessment scale for included studies is used to assessing the quality of included articles to see which study is methodologically better than the other one and how a low-quality article can impact on the main result.

To conduct an assessment of the quality of the included articles, we employed the Newcastle–Ottawa (NCO) Quality Assessment Scale Checklist, as depicted in Fig. 1. This checklist comprises three sections: Selection, Comparability, and Exposure. Each part aimed at scrutinizing the overall quality of the studies and identifying potential errors.

### Statistical analysis

We employed Comprehensive Meta-Analysis (CMA) software version 3 as our analytical tool. In our analysis, we utilized odds ratio (OR) with 95% confidence intervals to assess the influence of family history of cancer on CRC risk, as reported in the included studies. To assess the degree of heterogeneity, we employed the *I2* statistic, considering significance at a level greater than 50%. In instances of significant heterogeneity, we applied a random effects model. To delve deeper into the sources of heterogeneity, we conducted subgroup analyses based on the degree of evaluated relatives. Additionally, we used meta-regression to explore potential causes of heterogeneity, considering factors such as sample size and the Human Development Index (HDI). For an assessment of potential publication bias, we employed a funnel plot. Finally, power analysis was utilized to evaluate whether the mean sample size was sufficient for our study's objectives.

## Results

### Aim and trends

The primary objective of our present study is to explore the potential influence of a positive family history of cancer in patients with CRC within the EMRO. Through statistical analysis, we have identified a significant correlation between this risk factor and the incidence of CRC.

### Description of search

**Figure 2:**
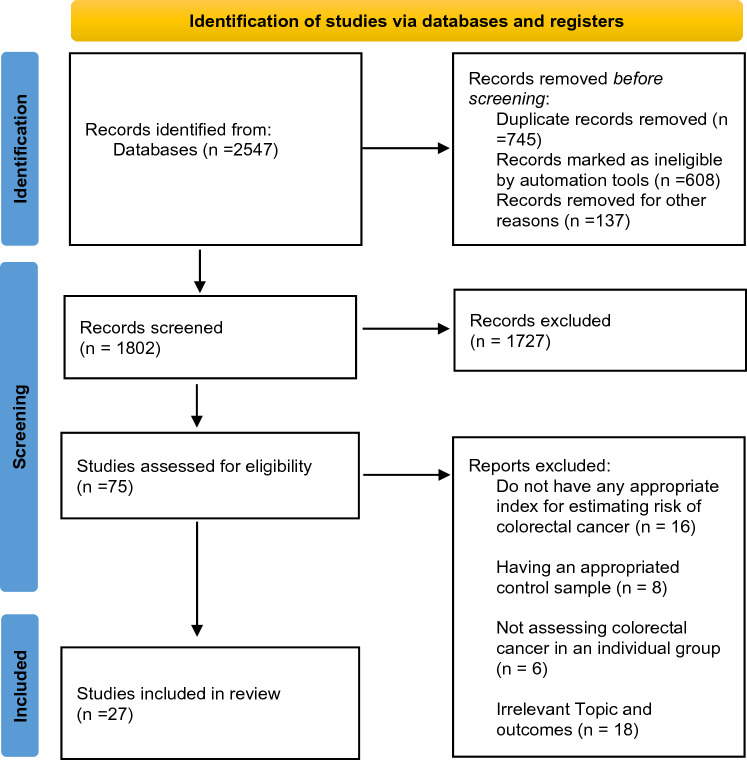
Flow chart of study selection process to showed that all of our study selection process is done under the guidelines of Preferred Reporting Items for Systematic Reviews and Meta-Analysis.

We have meticulously documented our systematic search process in accordance with the PRISMA checklist guidelines, as illustrated in Fig. 2. Our search extended up to December 2022 and covered the databases mentioned above. After eliminating duplicate entries, we identified 1802 articles for initial screening based on their titles and abstracts. Following the exclusion of irrelevant articles, we subjected 75 to a thorough full-text review. Ultimately, we carefully selected 27 articles for inclusion in our meta-analysis.

### Characteristics of the included studies

Table 1 provides an overview of the included papers in our analysis. Our meta-analysis comprises 27 studies conducted in EMRO countries, encompassing a total of 13,939 patients. Among these studies, 10 originated from Iran, five from Jordan, and two each from Saudi Arabia, Egypt, and Morocco. Kuwait, Palestine, Oman, Tunisia, Qatar, and Pakistan each contributed one study to our analysis.

**Table 1:** Main characteristics of included studies.

Author, Year	Country	Journal	Sample size	Male/Female	History of cancer	Number of patients with FH (case/control)	Significance of the results (presented by OR)	Quality assessment based on NCO scale
Tayyem, 2015^16^	Jordan	Nutrients	417	193/224	CRC Cancer Yes/No	69/90	(OR 1.21; 95% CI 0.81–1.81)	8
Shivappa,2017^17^	Iran	Asian Pacific Journal of Cancer Prevention	213	105/108	CRC First and Second Degree	13/3	(OR 10.38; 95% CI 2.85–37.81)	8
Alsheridah, 2018^18^	Kuwait	BMC Cancer	309	168/141	CRC Cancer Yes/No	18/22	(OR 1.77; 95% CI 0.90–3.47)	8
Alqahtani, 2020^19^	Saudi Arabia	Journal of Family Medicine and Primary Care	242	138/104	CRC Cancer Yes/No	19/9	(OR 2.31; 95% CI 1.00–5.35)	8
Deoula, 2020^20^	Morocco	JMIR research protocols	2966	1474/1492	CRC Cancer Yes/No	83/12	(OR 7.26; 95% CI 3.95–13.37)	7
Khatatbeh, 2018^21^	Jordan	Asian Pacific Journal of Cancer Prevention	300	147/153	CRC Cancer Yes/No	19/13	(OR 3.25; 95% CI 1.53–6.90)	8
Lo, 2010^22^	Egypt	Diseases of the Colon & Rectum	860	457/403	CRC Cancer Yes/No Any Cancer Yes/No	43/46	CRC: (OR 5.86; 95% CI 1.29–26.60)/ANY: (OR 0.97; 95% CI 0.62–1.50)	6
Ghrouz, 2021^23^	Palestine	Nutrition and Cancer	210	115/95	CRC Cancer Yes/No, Any Cancer Yes/No	22/5	CRC: (OR 4.82; 95% CI 1.01–22.91)/ANY: (OR 5.30; 95% CI 1.92–14.60)	7
Soliman, 1998^24^	Egypt	International journal of cancer	222	120/102	CRC Cancer Yes/No Any Cancer Yes/No	18/1	CRC: (OR 8.54; 95% CI 1.05–69.50)/ANY: (OR 21.29; 95% CI 2.78–162.51)	5
Azzeh 2017^25^	Saudi Arabia	BMC Public Health	301	NA	CRC Cancer Yes/No	22/27	(OR 0.97; 95% CI 0.52–1.79)	6
Farahani 2020^26^	Iran	Journal of Gastrointestinal Cancer	170	90/80	CRC Cancer Yes/No	21/8	(OR 3.44; 95% CI 1.42–8.29)	6
El kinany, 2020^27^	Morocco	European Journal of Nutrition	2906	1432/1474	CRC Cancer Yes/No	80/12	(OR 6.99; 95% CI 3.79–12.89)	9
Mafiana, 2018^28^	Oman	Asian Pacific Journal of Cancer Prevention	279	141/138	CRC Cancer Yes/No	97/161	(OR 0.45; 95% CI 0.18–1.11)	8
Abu Mweis, 2015^29^	Jordan	European Journal of Cancer Prevention	407	187/220	CRC Cancer Yes/No	68/89	(OR 1.16; 95% CI 0.77–1.74)	6
Gharbi, 2020^30^	Tunisia	La Tunisie Médicale	102	52/50	CRC Cancer Yes/No	13/7	(OR 2.15; 95% CI 0.77–5.94)	7
Teimoorian, 2018^31^	Iran	Iranian Journal of Pathology	117	NA	CRC Cancer Yes/No, Any Cancer Yes/No	1/4	CRC: (OR 0.49; 95% CI 0.02–9.38)/ANY: (OR 1.50; 95% CI 0.15–14.29)	6
Tayyem, 2016^32^	Jordan	Integrative cancer Therapies	501	248/253	CRC Cancer Yes/No	84/101	(OR 1.10; 95% CI 0.76–1.58)	6
Arafa, 2011^33^	Jordan	Asian Pacific Journal of Cancer Prevention	440	236/204	CRC First Degree	20/9	(OR 2.34; 95% CI 1.04–5.27)	6
Azizi, 2015^34^	Iran	Asian Pacific Journal of Cancer Prevention	414	220/194	CRC First Degree	58/28	(OR 2.48; 95% CI 1.50–4.10)	7
Rafiee, 2020^35^	Iran	European Journal of Cancer Prevention	370	199/171	CRC First Degree, Any Cancer Yes/No	66/89	CRC: (OR 0.73; 95% CI 0.22–2.37)/ANY: (OR 1.75; 95% CI 1.13–2.69)	6
Bener, 2010^36^	Qatar	Asian Pacific Journal of Cancer Prevention	428	149/179	Any Cancer Yes/No	61/52	(OR 3.17; 95% CI 2.03–4.95)	6
Safaee, 2010^37^	Iran	Indian Journal of Cancer	786	424/362	Any Cancer Yes/No	143/96	(OR 1.77; 95% CI 1.30–2.40)	7
Golshiri, 2016^38^	Iran	International Journal of Preventive Medicine	200	122/78	Any Cancer Yes/No	34/36	(OR 0.91; 95% CI 0.51–1.63)	8
Moazzen, 2020^39^	Iran	Annals of Global Health	405	0/405	Any Cancer Yes/No	33/49	(OR 1.45; 95% CI 0.88–2.40)	9
Khan, 2015^40^	Pakistan	Asian Pacific Journal of Cancer Prevention	222	105/117	Any Cancer Yes/No	14/14	(OR 2.23; 95% CI 1.00–4.97)	9
Abolhassani, 2019^41^	Iran	Ecotoxicology and Environmental Safety	72	43/29	Any Cancer Yes/No	12/9	(OR 0.93; 95% CI 0.33–2.61)	7
Khodaverdi, 2021^42^	Iran	BMC Cancer	80	40/40	Any Cancer Yes/No	4/3	(OR 1.37; 95% CI 0.28–6.55)	6

*FH* family history, *OR* odd ratio, *NCO* Newcastle–Ottawa, *CRC* colorectal cancer, *NA* not available.

All of the selected articles investigated the disparities in the presence of a positive family history of cancer between case and control groups. The publication dates of the included articles span from 1998 to 2021. The mean sample size across these studies was 516, ranging from 72 to 2966.

### Main result

**Figure 3:**
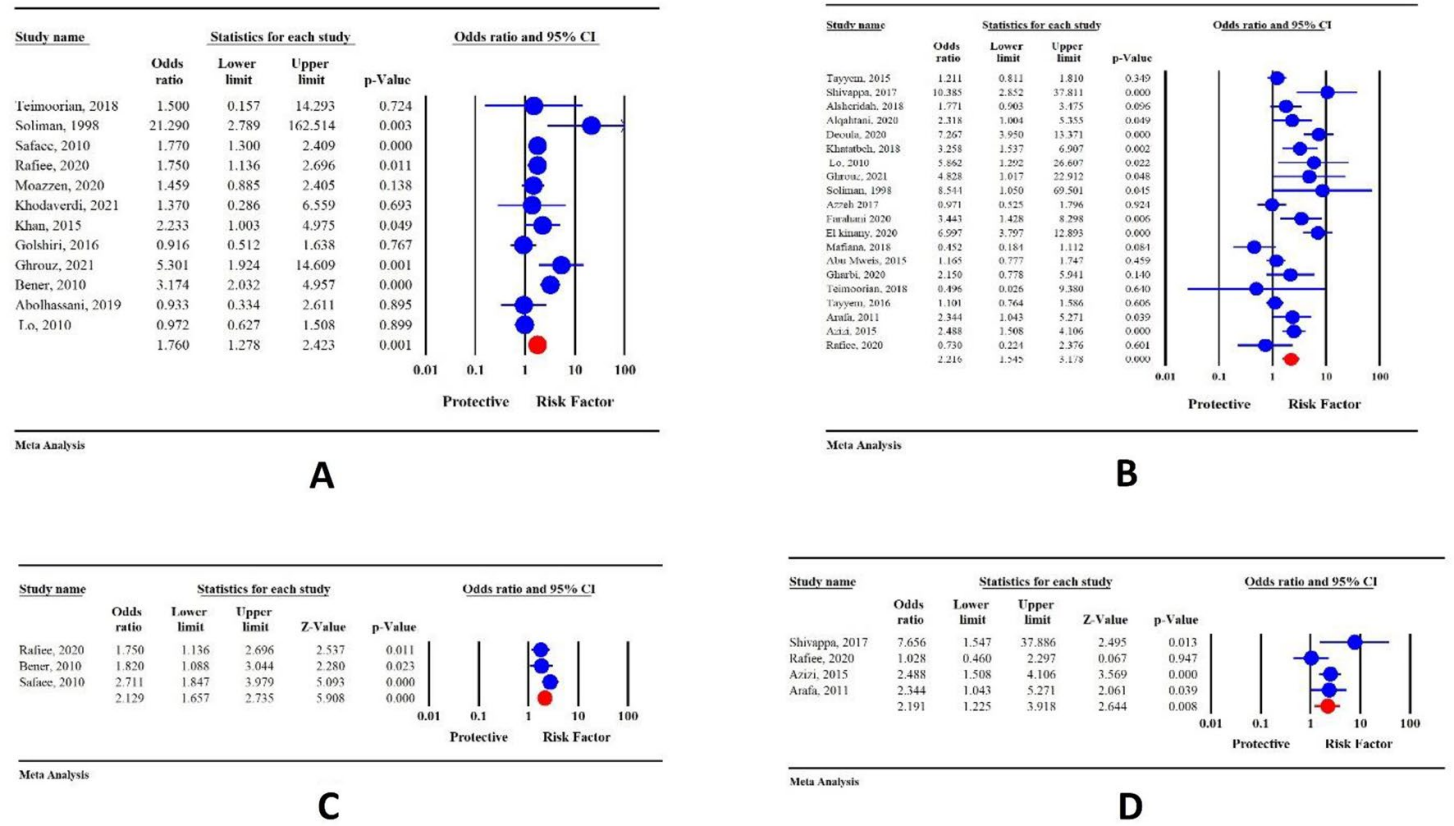
(**A**) Forest plot for the relationship between positive family history of any cancer and colorectal cancer is performed to see if there is a positive relationship between a positive family history of any cancer and risk of colorectal cancer or not, (**B**) Forest plot for the relationship between positive family history of CRC cancer and colorectal cancer performed to see if there is a positive relationship between a positive family history of CRC and risk of colorectal cancer or not, (**C**) Forest plot for the relationship between positive family history of any cancer in first-degree relatives and colorectal cancer is performed to see people having a positive first-degree family history of any cancer can experience a higher risk of colorectal cancer than other types of positive family history of any cancer or not, (**D**) Forest plot for the relationship between positive family history of CRC in first-degree relatives and colorectal cancer is performed to see people having a positive first-degree family history of CRC can experience a higher risk of colorectal cancer than other types of positive family history of CRC or not.

Our meta-analysis showed a significant association between the presence of a family history of CRC or any cancers with CRC (OR 2.21; 95% CI 1.54–3.17; P < 0.001 and OR 1.76; 95% CI 1.27–2.42; P = 0.001, respectively) (Fig. 3A,B).

### Subgroup analysis

Obtained results from subgroup analysis revealed that positive family history of any cancers in the first-degree relatives significantly increases the risk of CRC (OR 2.12; 95% CI 1.65–2.73; P < 0.001) (Fig. 3C). A positive family history of CRC in first-degree relatives is also associated with an increased risk of CRC (OR 2.19; 95% CI 1.22–3.91; P = 0.008) (Fig. 3D).

### Sensitivity and power analysis

**Figure 4:**
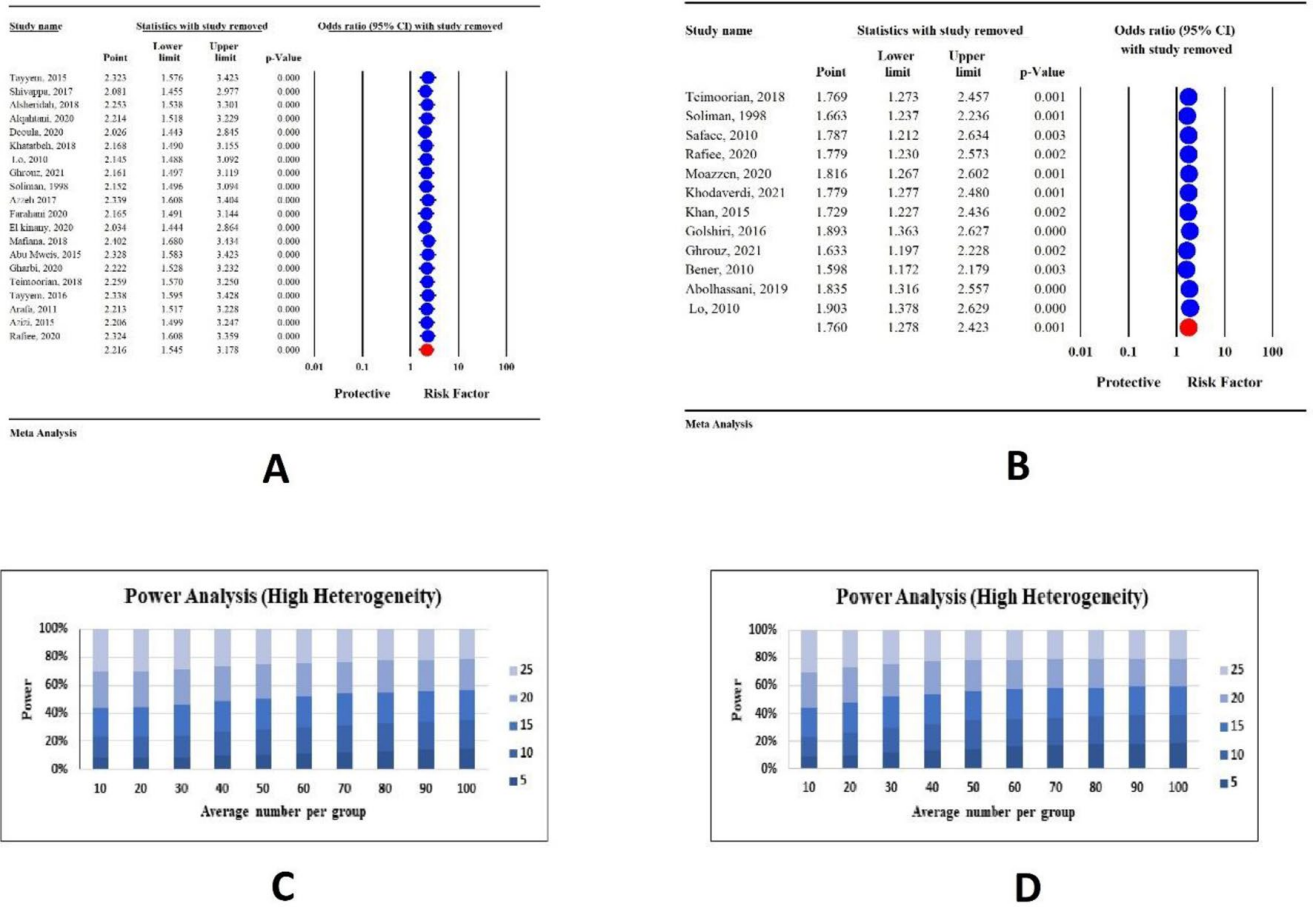
(**A**) Result of sensitivity analysis by one study removing in CRC group to find the potential source of heterogeneity, (**B**) Result of sensitivity analysis by one study removing in any cancer group to find the potential source of heterogeneity, (**C**) Result of power analysis to find the source of heterogeneity in CRC relationship, (**D**) Result of power analysis to find the source of heterogeneity in any cancer relationship.

Due to the observed heterogeneity in results, we performed a sensitivity analysis utilizing the 'one study removal' approach to identify potential sources of this heterogeneity. The results of the sensitivity analysis demonstrated that the primary outcome remained consistent, with no significant alterations observed in both CRC and any any cancer group when any single study was excluded (Fig. 4A,B).

Additionally, the result of Power analysis showed that the mean number of sample sizes for CRC and any cancer were adequate for the meta-analysis (Fig. 4C,D).

### Result of meta-regression

**Figure 5:**
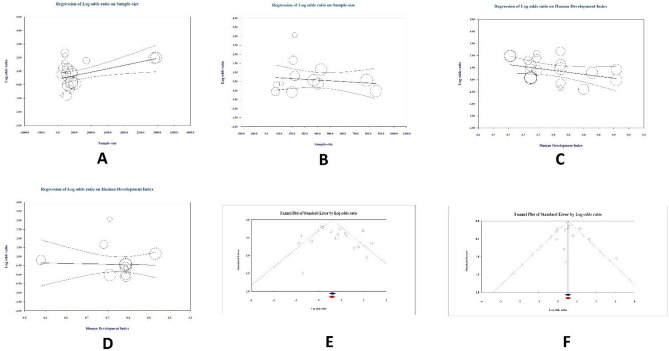
(**A**) Result of meta regression based on number of sample size in CRC group. This analysis is performed to see if there is a relationship between increasing number of sample size and risk of CRC among positive family history of CRC, (**B**) Result of meta regression based on number of sample size in any cancer group. This analysis is performed to see if there is a relationship between increasing number of sample size and risk of CRC among positive family history of any cancer, (**C**) Result of meta regression based on level of human developmental index (HDI) in CRC group. This analysis is performed to see if there is a relationship between increasing the rate of HDI and risk of CRC among positive family history of CRC, (**D**) Result of meta regression based on level of HDI in any cancer group. This analysis is performed to see if there is a relationship between increasing the rate of HDI and risk of CRC among positive family history of any cancer, (**E**) Funnel plot for assessing the source of heterogeneity in CRC group. This analysis is performed to find the source of Heterogeneity, (**F**) Funnel plot for assessing the source of heterogeneity in any cancer group. This analysis is performed to find the source of Heterogeneity.

The results of our meta-regression analysis, based on sample size, indicated a significant influence of increased sample size on the impact of a positive family history of CRC on the development of CRC (Coefficient = 0.0005, P = 0.001). Nevertheless, the cumulative effect of a positive family history of any cancer was not found to be influenced by an increase in sample size (Coefficient = − 0.0004, P = 0.538) (Fig. 5A,B).

We also conducted a meta-regression analysis to evaluate the influence of the HDI level in each study on our final results^43^. We did not find a significant effect of HDI on the relation between positive family history of CRC and CRC development (Coefficient = − 5.9501, P = 0.057). In addition, our investigations did not show a significant effect of HDI on the positive family history of CRC and any other cancer on CRC development (Coefficient = − 0.3850, P = 0.868) (Fig. 5C,D).

### Publication bias

We employed a funnel plot to evaluate potential publication bias. Our primary findings revealed no significant publication bias for both any cancer and CRC (Egger's test P = 0.52; Begg's test P = 0.73, Egger's test P = 0.13; Begg's test P = 0.38, respectively) (Fig. 5E,F).

## Discussion

CRC, one of the most prevalent forms of cancer, plays a significant role in global health, leading to numerous fatalities and a substantial burden of morbidity^1^. Numerous epidemiological studies have assessed the risk of CRC among individuals with a family history of this condition^44^. CRC is influenced by a combination of environmental and hereditary factors, which are recognized as risk factors for the disease^45^. Furthermore, individuals with inherited conditions such as HNPCC and Familial adenomatous polyposis, as well as those with a positive family history of CRC among their relatives, face an elevated risk of developing this condition^46^.

Our study revealed a robust correlation between a positive family history of CRC and an increased risk of CRC within the EMRO region. A study conducted by Ramírez-Díaz further confirmed the crucial role of family history as a significant factor influencing the occurrence of CRC^47^. These findings may be attributed to shared or similar gene sequences among family members, which could accelerate the mechanisms of CRC. Furthermore, a systematic review conducted by Henrikson et al. confirmed these results, demonstrating that a positive family history of CRC increases the risk of developing this condition^48^. In addition, the Slattery et al. study identified a positive association between individuals having a positive family history of CRC and an elevated risk of developing CRC^49^.

Several well-established gene mutations are associated with the development of CRC, and these mutations can be categorized into different classes of genes^50^. Familial adenomatous polyposis serves as an exemplar of a mutation occurring in a tumor suppressor gene, specifically in the APC gene^51^. Another similar polyposis syndrome related to CRC development is MUTYH (MYH)-associated polyposis (MAP) which is caused by a DNA repair gene alteration named MYH^52^. Lynch syndrome also known as HNPCC is a common predisposing condition of CRC accounting for 2–5% of all cases, and is a result of mutation in DNA mismatch repair genes mainly affecting MLH1, MSH2, and MSH6.4^53^.

Our subgroup analysis revealed a notable disparity in CRC incidence within the EMRO region. Specifically, individuals with first-degree relatives affected by CRC were more than twice as likely to develop CRC compared to those without a family history of the disease. This observed risk is significantly higher than the findings reported in Fuchs et al.'s study, which encompassed a prospective study of over 100,000 volunteers in the USA. Remarkably, their investigation concluded that having a first-degree family member with a positive history of CRC could elevate an individual's risk of developing CRC by a substantial 72%. These findings emphasize the importance of familial predisposition as a significant risk factor for CRC within the EMRO region and warrant further exploration and targeted preventive measures^8^. In a meta-analysis conducted by Adam S., it was found that individuals with at least one positive first-degree family history have a heightened risk of developing CRC^54^. In a study conducted by Mehraban Far, it was established that a positive family history of CRC in first-degree relatives significantly amplified the risk of developing CRC^55^. Another noteworthy result from our meta-analysis indicated that having a family history of any type of cancer also increases the risk of developing CRC.

One of the most significant findings from our analysis is that a family history of CRC is more strongly associated with an increased risk of CRC in the EMRO region than having a positive family history of any cancer. These disparities can be attributed to several factors. Certain cancers may be less likely to be inherited or influenced by genetics. Newschaffer et al. demonstrated that a positive history of breast cancer did not elevate the risk of CRC and, in some subgroups, could even be considered a protective factor^56^. In a recent study conducted by Beebe-Dimmer et al., it was found that a positive family history of prostate cancer did not result in a significant increase in the risk of developing CRC^57^.

EMRO region people may have a higher susceptibility for CRC than other cancers due to genetical reasons. Within this context, several studies have pinpointed various genes as potential contributors to the risk of CRC development. These genes may vary in prevalence between populations, potentially explaining the differing effect sizes of a positive family history of CRC on increased risk of CRC between our study and others. In a study carried out by Dunlop et al., it was revealed that the median count of risk alleles was higher in cases than in controls^58^. Furthermore, Yusuf et al. identified several replicated associations as genetic risk factors for CRC^59^.

Environmental factors and dietary habits in this region may also contribute to an elevated risk of CRC in this region. According to a meta-analysis, smoking emerged as a significant risk factor for CRC in the EMRO region^60^. Seyyedsalehi et al., through a multicenter case–control study in Iran, identified a significant positive association between CRC and high dietary total fat intake^61^.

However, these results could also be due to better CRC screening in this region to identify suspicious and possible cases for early treatment. It was shown that due to a recent surge in early-onset diagnoses in the USA, approximately 10% of CRC cases are now detected in individuals under the age of 50^62^.

Contrary to the good screening of CRC, some cancers may not be diagnosed well until the final stages, and this lack of timely diagnosis can be one of the reasons for the lower percentage of the risk factor of any cancer compared to CRC. For instance, statistics show that in the USA, one-third of cervical cancers are diagnosed in the late stages when treatment is more difficult^63^.

Lastly, our meta-analysis reveals a compelling discovery: the substantial influence of a first-degree family history of any cancer group on the heightened susceptibility to CRC. This finding sounds a resounding alarm particularly for individuals with this positive familial heritage. It emphasizes the urgency of proactive measures. Timely detection and intervention in cases of CRC can yield remarkable results, primarily in terms of preventing mortality and morbidity.

### Limitations

Our study has several limitations. Firstly, some countries in the EMRO region lacked sufficient relevant articles to be included in our study. Secondly, we lacked information regarding family histories of other inheritable cancer types, potentially limiting our comprehensive understanding of inherited cancer risks. Thirdly, we lacked sufficient data to explore whether a positive history of cancer among second- or third-degree relatives could be a potential risk factor. Lastly, the included studies did not provide information on the specific types of cancer that affected the family members, which could have added valuable context to our findings.

## Conclusion

In summary, our meta-analysis conclusively establishes a significant positive association between having a family history of CRC or any cancer and the heightened risk of developing CRC. Our study underscores the critical importance of prioritizing screening and early identification for individuals with a family history of malignancies. By fostering collaboration among healthcare facilities and promoting the utilization of screening methods for timely detection, we can substantially mitigate the burdens of morbidity, mortality, and financial costs borne by both governments and the general public. Moreover, individuals with a positive family history should seek consultation with specialists for the screening tests and examinations to facilitate early diagnosis and appropriate treatment.

## Data Availability

All data analyzed during this study are included in this published article.
